# An injured tissue affects the opposite intact peritoneum during postoperative adhesion formation

**DOI:** 10.1038/srep07668

**Published:** 2015-01-08

**Authors:** Tatsuya Suzuki, Toru Kono, Hiroki Bochimoto, Yoshiki Hira, Tsuyoshi Watanabe, Hiroyuki Furukawa

**Affiliations:** 1 Division of Gastroenterologic and General Surgery, Department of Surgery, Asahikawa Medical University, Hokkaido, Japan; 2 Advanced Surgery Center, Sapporo Higashi Tokushukai Hospital, Hokkaido, Japan; 3 Faculty of Pharmaceutical Sciences, Hokkaido University, Hokkaido, Japan; 4 Department of Microscopic Anatomy and Cell Biology, Asahikawa Medical University, Hokkaido, Japan

## Abstract

The pathophysiology of adhesion formation needs to be clarified to reduce the adhesion-related morbidity. The epithelial characteristics of the peritoneum suggest a protective role against adhesion formation, yet how the peritoneum is involved in adhesion formation is not well characterized. We microscopically observed an experimental model of adhesion formation to investigate the effects of an injured tissue on the opposite intact peritoneum. Adhesions were induced between injured and intact hepatic lobes, and the intact peritoneum opposite to the injured tissue was examined for 8 days. The opposite intact peritoneum was denuded of mesothelial cells for 6 hours, and the remnant mesothelial cells changed morphologically for 24 hours. The detachment of mesothelial cells allowed fibrin to attach to the basement membrane of the opposite peritoneum, connecting the two lobes. Moreover, macrophages and myofibroblasts accumulated between the two lobes, and angiogenesis occurred from the opposite intact lobe to the injured lobe. These observations indicate that an injured tissue deprives the opposite intact peritoneum of its epithelial structure and causes fibrous adhesions to the opposite intact tissue. This study implies a possible role of mesothelial cells for barrier function against adhesion formation, that is, keeping mesothelial cells intact might lead to its prophylaxis.

Postoperative adhesions after abdominopelvic surgery cause small-bowel obstruction, female infertility and inadvertent organ injury during adhesiolysis at reoperation, and the large impact of adhesion-related morbidity on human health and healthcare costs emphasizes the need to develop prophylactic strategies^1^,^2^,^3^. Although various prophylactic agents have been developed and shown to be effective in adhesion prevention, limited clinical trials have shown efficacy in reduction of adhesion-related morbidity^4^,^5^,^6^. A better understanding of the pathophysiology of adhesion formation is essential to improving prophylactic strategies and reducing adhesion-related morbidity.

The process of adhesion formation is briefly described as follows: tissue injury involving the peritoneum causes inflammation and fibrin deposition, which connects the injured tissue to the tissue of an opposing organ. If the fibrin persists due to suppressed fibrinolysis, tissue repair extends into the fibrin scaffolding, leading to scar formation^5^,^7^,^8^. The mechanisms of the fibrinous connection of tissues, such as inflammation and fibrinolysis, have been examined in detail^9^,^10^,^11^,^12^. However, the role of the peritoneum in adhesion formation has not been well studied.

The peritoneum may act as a protective epithelium against adhesion formation, as suggested by the fact that the peritoneum consists of mesothelial cells (MCs) and a basement membrane (BM) and displays epithelial characteristics^13^. Schade and Williamson noted that detachment of MCs appeared to be the critical event in an experimental adhesion model^14^. Moreover, Lamont et al. and Haney and Doty showed that adhesions more frequently formed when two opposing peritoneums were injured than when only one peritoneum was injured while the opposite peritoneum was left intact^15^,^16^. These studies suggest that adhesions form only after both of the two opposing peritoneums lose their epithelial structure.

Little is known, however, about how an injured tissue affects the opposite intact peritoneum when these tissues are approximated. Only one side of the two opposing peritoneums is intentionally injured in many experimental adhesion models^9^,^10^,^11^, which implies that adhesions form when an injured tissue and the opposite intact peritoneum are approximated. Hence, we hypothesized that an injured tissue would deprive the opposite intact peritoneum of its epithelial structure and form adhesions with the opposite intact tissue.

In this study, we performed a histological examination of an experimental model of adhesions between injured and intact hepatic lobes to clarify how an injured tissue affects the opposite intact peritoneum during adhesion formation.

## Results

### Adhesions form between a cauterized lobe and the opposite intact lobe of the rat liver

**Figure 1:**
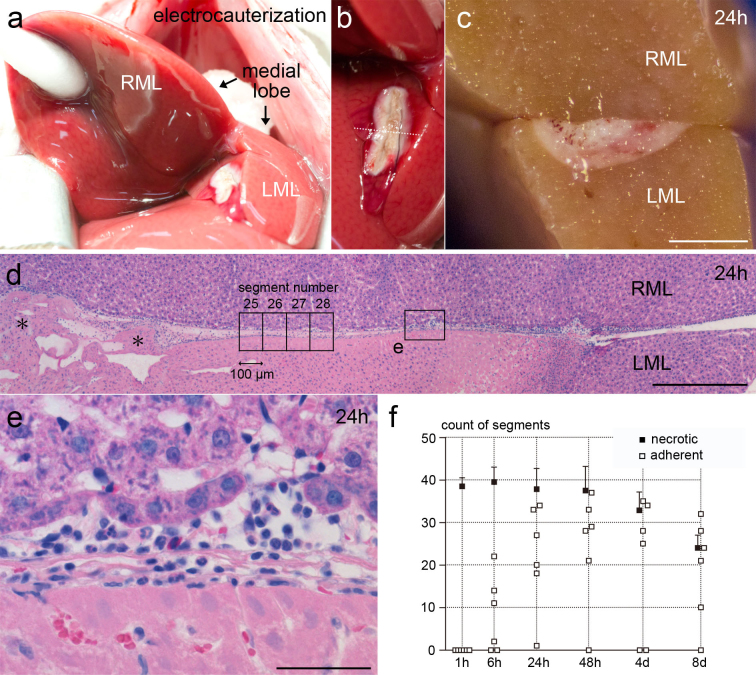
Hepatic model of adhesion formation. (a,b) Photographs of the liver at the time of electrocauterization. (c) A cross section of the excised liver at 24 hours. Bar, 2 mm. (d) HE staining of the adherent lobes at 24 hours. An adhesion is seen over the necrotic hepatocytes, which are eosinophilic. The surface is uneven in the central part of the cauterized area (asterisks). Micrographs were divided into 100-μm-wide segments. Bar, 400 μm. (e) A closer view of (d). Mononuclear cells and granulocytes are seen in the adherent area. Bar, 40 μm. (f) The counts of the segments in which hepatocytes were necrotic are expressed as the mean + s.d. (n = 6), whereas the counts of the segments in which the two lobes were adherent are shown in each value.

By HE staining, we observed that adhesions formed when a cauterized lobe and the opposite intact lobe were approximated. In this model, the cauterized tissue was severely injured, as indicated by the extensive necrosis of cauterized hepatocytes (Figure 1d), measuring 3833 ± 520 μm in width and 1617 ± 279 μm in depth at 24 hours (n = 6), and tissue deformation in the central part of the cauterized area. In contrast, the hepatocytes of the opposite lobe were intact in most of the area, except that focal necrosis was occasionally observed at 24 hours. Adhesions formed over the necrotic tissue (Figure 1d). Inflammation was conspicuous at 24 hours (Figure 1e), and tissue repair was proceeding at 4 days. The adherent area enlarged until 24 hours and did not change afterward, whereas adhesions were occasionally absent at 48 hours and later (Figure 1f). The necrotic area diminished at 8 days as tissue repair progressed.

All animals survived the surgery. Of 12 animals at each time point, including animals examined by electron microscopy, the incidence of adhesions, as assessed by gross and histological findings, was as follows: n = 0 at 1 hour, n = 8 at 6 hours, n = 12 at 24 hours, n = 9 at 48 hours, n = 7 at 4 days, and n = 9 at 8 days. No adhesions were present after sham operation (n = 3 at 24 hours and 4 days). These observations indicated that adhesions could form after approximation of a cauterized lobe and the opposite intact lobe. The findings described hereafter will pertain to adherent specimens at 48 hours after cauterization and later.

### The normal liver peritoneum is an epithelial tissue consisting of MCs and a BM

**Figure 2:**
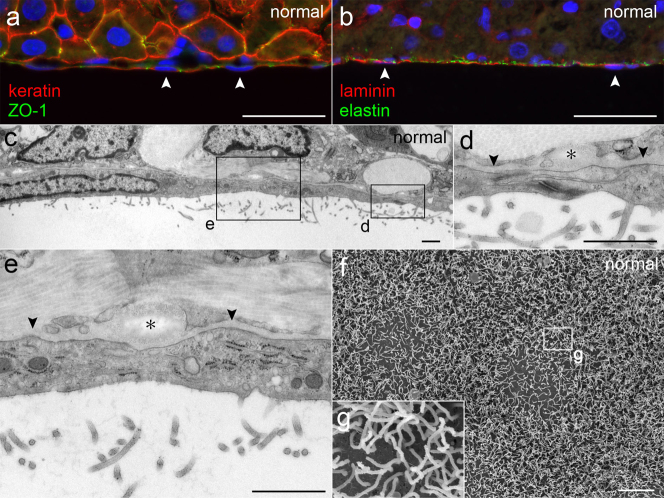
Normal liver peritoneum. (a,b) Immunohistochemical staining showing MCs having keratin and ZO-1 and a layer of laminin and elastin beneath MCs. The nuclei of MCs (arrowheads) are indicated. Bar, 40 μm. (c–e) Micrographs of TEM showing flat MCs, a cell-cell junction and microvilli. The BM (arrowheads) is a thin layer of moderate density. Elastic fibers (asterisks) are lucent with fine higher density at periphery. Bar, 1 μm. (f,g) Micrographs of SEM showing the microvilli of MCs. Bar, 5 μm.

Examination of the normal liver peritoneum showed that this tissue possessed typical epithelial characteristics (Figure 2). Normal MCs had numerous microvilli, were present on the BM, and were flat and connected to each other by cell-cell junctions. Beneath the BM, a layer of elastic fibers was present. The elastic fibers were thicker and more distinct than the BM and are indicated in the micrographs to show the peritoneal layer of the opposite lobe.

### The peritoneum of the opposite lobe is denuded of MCs until 6 hours

**Figure 3:**
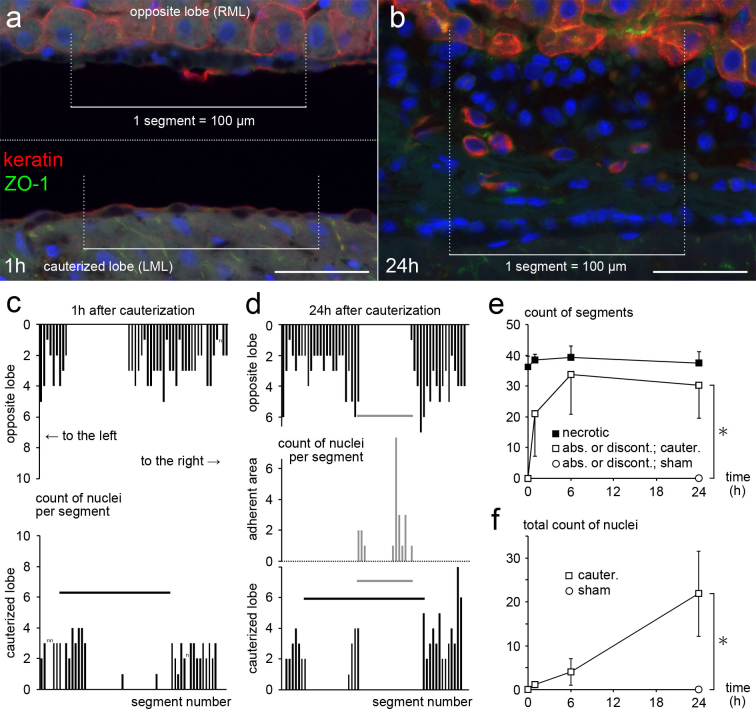
Distribution of MCs and their quantification. (a,b) Immunohistochemical staining for keratin and ZO-1 at 1 hour showing an edge of the denuded peritoneum, and at 24 hours showing a segment in which isolated MCs are remarkably seen in the adherent area. Bar, 40 μm. (c,d) Representative histograms of the counts of MCs per segment at 1 hour and 24 hours. Black horizontal bars indicate the cauterized area, and gray bars indicate the adherent area, in which the isolated MCs were present. (e) The counts of the segments in which the MCs of the opposite lobe were absent or discontinuous (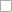
after cauterization, 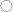
after sham operation), and the counts of the segments in which hepatocytes of the cauterized lobe were necrotic (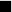) are shown. (f) The total counts of nuclei of the isolated MCs in the entire section. (e,f) The values at immediately after cauterization are shown at 0 hour. Values are expressed as the mean ± s.d. (n = 4 at immediately after cauterization; n = 6 at 1, 6 and 24 hours after cauterization; and n = 3 at 24 hours after sham operation) *P < 0.05 by Mann-Whitney's U test.

We examined the early effects of a cauterized tissue on the opposite peritoneum. MCs were counted to determine their distribution and quantify their presence (Figure 3). As shown in the representative histograms, the MCs of the opposite lobe began decreasing in number until 1 hour and disappeared in continuous segments (Figure 3c). More than half of the opposite peritoneum that came into contact with the necrotic tissue had lost MCs at 1 hour (Figure 3e). The denuded area appeared to enlarge until 6 hours and did not change at 24 hours (Figure 3e).

**Figure 4:**
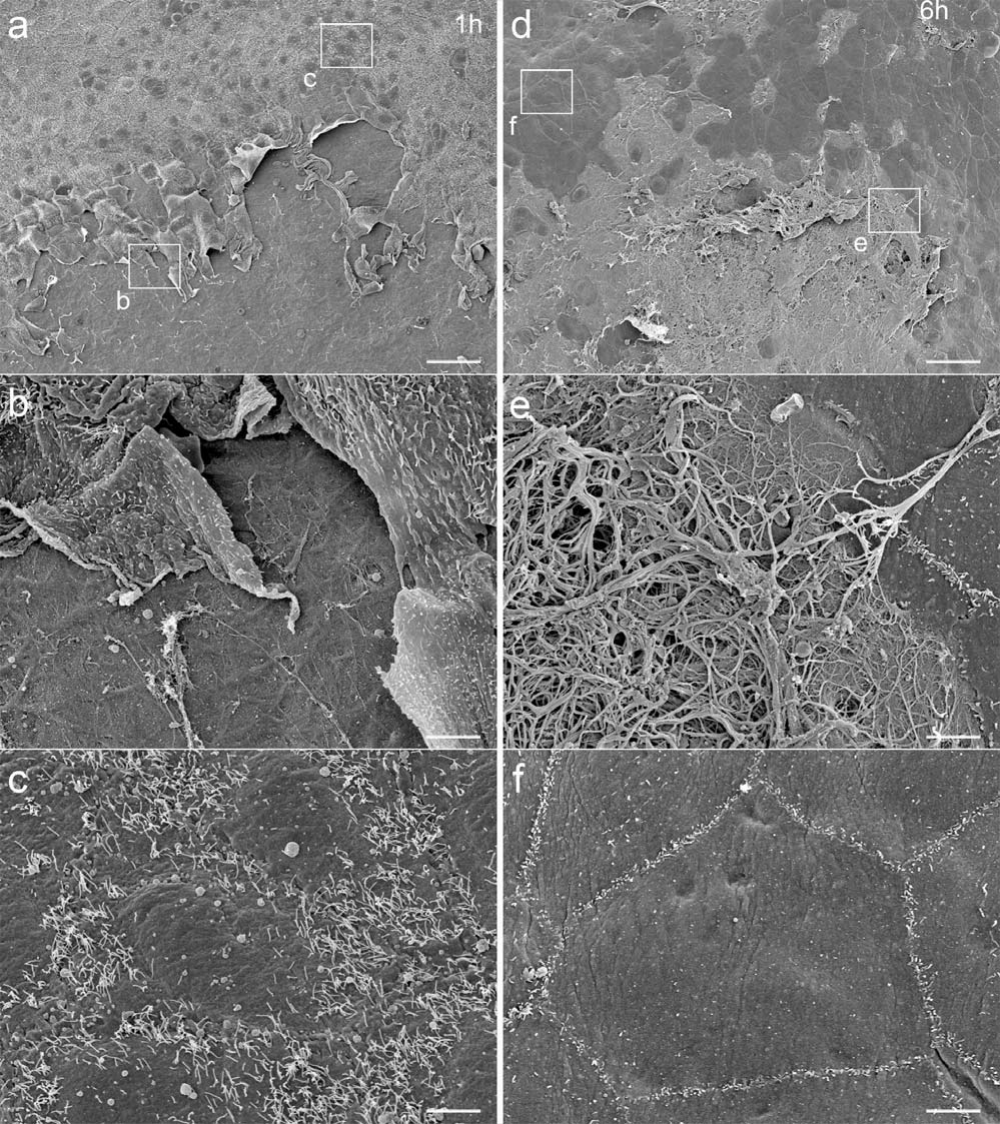
Observation of opposite peritoneum by SEM. (a–c) At 1 hour, the opposite peritoneum has been denuded of MCs, leaving an island-shaped denuded area, where the underlying BM is exposed. The MCs at the margin are irregularly shaped. Microvilli are fewer around the margin. (d–f) At 6 hours, fibrin fibers are seen over the denuded area. The MCs around the margin have much fewer and shorter microvilli. Bar, (a,d) 50 μm, (b,c,e,f) 5 μm.

When observed by electron microscopy, the opposite peritoneum was denuded of MCs in a large island-shaped area at 1 hour, revealing the underlying BM (Figures 4a and b). The irregular surfaces of the MCs at the margin of the denuded area (Figures 4a and b) and swollen cytoplasmic organelles observed by TEM (data not shown) indicated that these cells were severely injured. At 6 hours, the exposed BM was often covered by fibrin (Figures 4d and e). The injured MCs observed at the margin at 1 hour were rarely observed (Figures 4d and e), whereas isolated and severely injured MCs and necrotic debris were occasionally observed under the covering fibrin by TEM (data not shown). This finding suggested that the injured MCs at the margin were detached and more severely injured until 6 hours. In contrast, remnant MCs around the margin notably reduced the number of their microvilli until 6 hours (Figures 4a, c, d, and f), yet they possessed normal cytoplasmic organelles at 6 hours (data not shown), suggesting that the cells' injuries were mild and reversible.

In the rats for controls, no peritoneal denudation was observed in the opposite peritoneum immediately after cauterization (Figure 3e), showing that neither of the procedures of lifting the RML with a cotton swab nor cauterizing the LML induced detachment or injuries of MCs on the opposite peritoneum at the time of surgery. Similarly, no peritoneal denudation in the opposite peritoneum at 24 hours after sham operation (Figure 3e) indicates that the denudation at 24 hours after cauterization was caused by the cauterized tissue itself, and not by the surgical procedures other than cauterization. Also, the microvilli of MCs did not decrease in number at 6 hours after sham operation when observed by SEM.

These findings indicated that the early effects of a cauterized tissue on the opposite intact peritoneum were the detachment and injury of MCs in a large area, leading to exposure of the underlying BM to deposited fibrin. The remnant MCs around the margin, in contrast, appeared to survive.

### Inflammation increases until 24 hours, and fibrin attaches to exposed BM

**Figure 5:**
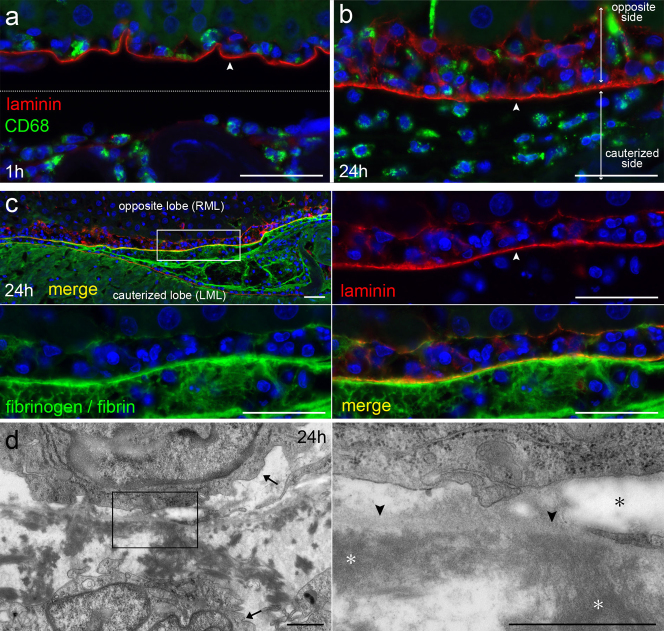
Inflammatory exudate increases until 24 hours, and deposited fibrin attaches to BM of opposite peritoneum. (a,b) Immunohistochemical staining for CD68 and laminin at 1 hour and 24 hours after cauterization. The cauterized lobe is in the lower part of the micrographs, and the opposite lobe is in the upper part. The layer of laminin (arrowhead) shows the BM of the opposite peritoneum. Bar, 40 μm. (c) Immunohistochemical staining for laminin and fibrinogen/fibrin at 24 hours. The layer of laminin (arrowhead) indicates the BM of the opposite peritoneum. Fibrinogen/fibrin is extensively seen between the cauterized tissue and the BM of the opposite peritoneum. Bar, 40 μm. (d) Micrographs of TEM at 24 hours. Fibrin (white asterisks) has attached to the BM (arrowheads) of the opposite peritoneum. The elastic fibers (black asterisk) are indicated. Macrophages (arrows) are seen on each side of the BM. Bar, 1 μm.

One of the noticeable events from 6 to 24 hours was infiltration of inflammatory cells into the adherent area. Macrophages did not increase in number at 1 hour, but appeared on the cauterized lobe and in the subserosa of the opposite lobe until 6 hours (Figure 5a). The presence of macrophages in the subserosa of the opposite lobe suggested that chemotaxis occurred through the peritoneal layer of the opposite lobe after the denudation of MCs. Neutrophils were also as conspicuous as macrophages on the cauterized lobe and in the subserosa of the opposite lobe at 6 hours (data not shown). The accumulation of macrophages increased until 24 hours (Figure 5b), whereas macrophages did not increase in number at 24 hours after sham operation (data not shown). These findings indicated that inflammation was most conspicuous at 24 hours and suggested that the cauterized tissue caused the chemotaxis of inflammatory cells from the opposite lobe to the cauterized lobe.

Another marked event until 24 hours was increasing deposition of fibrin. As observed by SEM, the fibrin covering the denuded peritoneum increased from 1 hour to 6 hours (Figures 4a, b, d, and e). Observation by immunohistochemistry showed that fibrin was extensively present in the adherent area between the cauterized tissue and the BM of the opposite peritoneum at 24 hours (Figure 5c). In addition, observation by immunohistochemistry and TEM confirmed that fibrin had attached to the exposed BM of the opposite peritoneum at 24 hours (Figures 5c and d). These observations showed that the increasing fibrin that was deposited in the adherent area connected the two lobes over a broader area until 24 hours via the attachment of fibrin to the exposed BM of the opposite peritoneum.

### Remnant MCs in the opposite lobe undergo morphological changes for 24 hours after electrocauterization

We then further examined how the remnant MCs of the opposite lobe were affected by a cauterized tissue from 6 to 24 hours. Immunohistochemical staining for keratin and ZO-1 showed that there were isolated MCs present in the adherent area at 24 hours (Figure 3b). There appeared to be more isolated MCs in the peripheral part of the adherent area than in the central part (Figure 3d, gray bars). Isolated MCs were sparse in the adherent area; the averages of the counts of nuclei per segment in the adherent area were 0.78 ± 0.45 at 24 hours as to isolated MCs (n = 6). Nonetheless, isolated MCs obviously increased in number until 24 hours (Figure 3f).

**Figure 6:**
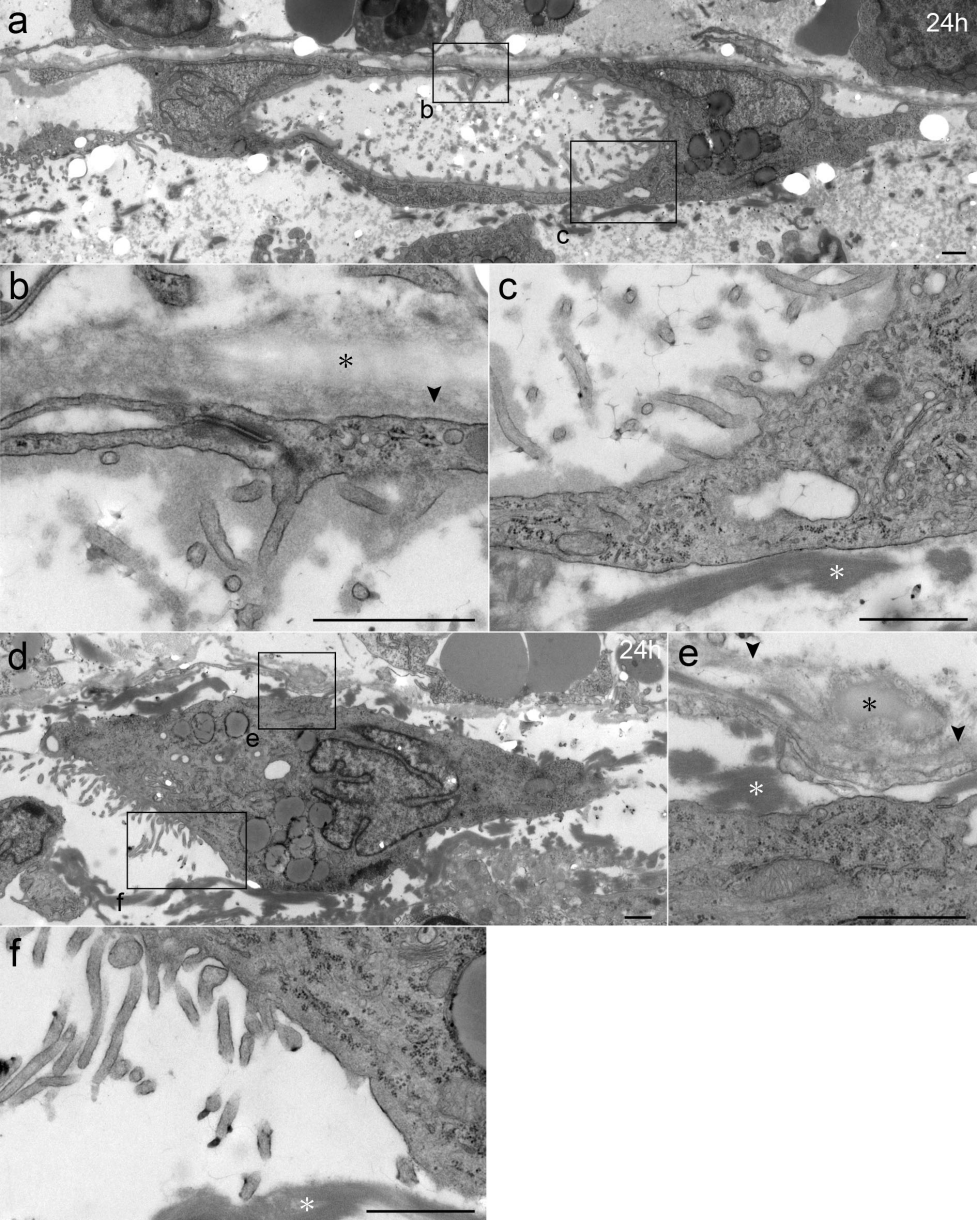
Micrographs of TEM showing isolated MCs in adherent area at 24 hours. (a–c) Saccular MCs are seen on the BM of the opposite peritoneum. Two nuclei are seen. (b) Cell-cell junctions are seen. The BM (arrowhead) and elastic fibers (black asterisk) of the opposite peritoneum are indicated. (c) The MCs have turned their microvilli inward, and their basal surfaces toward the surrounding fibrin. (d–f) The isolated and tall MCs in the adherent area at 24 hours. The MC is located away from the BM of the opposite peritoneum. It has detached from the BM and is in close proximity to fibrin. (f) The microvilli resemble those of normal MCs. Bar, (a–f) 1 μm.

Observation by TEM revealed isolated MCs in a saccular shape resting on the BM of the opposite peritoneum in the adherent area at 24 hours (Figure 6a). The cells were identified as MCs by their microvilli, which closely resembled those of normal MCs (Figure 6c, see also 2e). The saccular MCs were usually located on the BM of the opposite peritoneum (Figure 6b), indicating that these cells had originated from the opposite peritoneum. The saccular MCs were occasionally located away from the BM. Whereas several of the saccular MCs consisted of two MCs, others appeared to have been formed by a single MC. Fibrin was nearly invariably observed in close proximity to the outer rims of the saccular MCs (Figure 6c). Fibrin often attached to the exposed BM near the saccular MCs (data not shown).

Observation by TEM revealed another type of morphological change at 24 hours. There were isolated and tall MCs in the adherent area at 24 hours (Figure 6d). These cells were also identified as MCs by their microvilli (Figure 6f, see also 2e). Whereas several of these cells were located on the BM of the opposite peritoneum, others were located away from the BM and on fibrin, suggesting that the isolated and tall MCs on the opposite lobe had detached from the BM and were migrating into the adherent area. The isolated and tall MCs at 24 hours were presumed to originate from the opposite peritoneum because these cells often rested on that peritoneum's BM, whereas several of the cells appeared to have originated from the peritoneal margin of the cauterized lobe.

These observations showed that the remnant MCs of the opposite peritoneum underwent the two types of morphological changes in the adherent area until 24 hours after the procedure. We then observed how these affected MCs behaved afterward.

### Whereas several isolated MCs are still present in the adherent area at 48 hours and later, tall MCs regenerate the peritoneum on the marginal surfaces of the adherent area

Saccular MCs were present in the adherent area even at 48 hours and later. At 48 hours, there were two types of saccular MCs, i.e. those with very thin cytoplasm and scarce cytoplasmic organelles and those with thick cytoplasm and abundant organelles. At 4 days, only saccular MCs with thin cytoplasm were observed (data not shown).

In contrast, isolated and tall MCs were not observed inside the adherent area at 48 hours and later. Instead, relatively tall MCs were observed on the marginal surfaces of the adherent fibrin scaffolding and mostly formed cell-cell junctions with each other. This observation suggested that several of the isolated and tall MCs that had been observed at 24 hours participated in regeneration on the marginal surfaces. At 4 days, taller MCs with abundant cytoplasmic organelles nearly covered the marginal surfaces, forming a continuous peritoneum (data not shown).

### Tissue-repairing cells appear to migrate from the opposite intact lobe to the cauterized lobe

**Figure 7:**
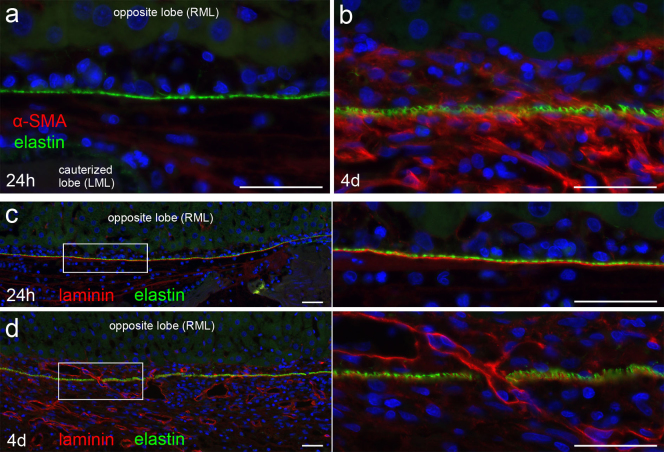
Tissue repair occurs between cauterized lobe and opposite intact lobe. (a,b) Immunohistochemical staining for α-SMA and elastin at 24 hours and 4 days. The cauterized lobe is in the lower part of the micrographs, and the opposite lobe is in the upper part. The layer of elastin shows the elastic fibers of the opposite peritoneum. Bar, 40 μm. (c,d) Immunohistochemical staining for laminin-γ1 and elastin at 24 hours and 4 days. A layer of the BM and elastic fibers of the opposite peritoneum is preserved at 24 hours. At 4 days, new layers of BM have appeared, forming lumen-like structures. The newly formed BM has grown through gaps of the elastic fibers of the opposite peritoneum. Bar, 40 μm.

Finally, we examined how a fibrous adhesion formed between a cauterized tissue and the opposite intact tissue. Immunohistochemical staining for α-SMA showed that myofibroblasts began infiltrating into the adherent area after 24 hours and obviously increased in number at 4 days (Figures 7a and b). The accumulation of myofibroblasts in the subserosa of the opposite lobe suggested that the cells were trying to migrate from the opposite lobe to the cauterized lobe. Myofibroblasts were not present in the subserosa of the opposite lobe at 4 days after sham operation (data not shown).

In addition, the opposite intact tissue appeared to contribute to angiogenesis in the adherent tissue. Immunohistochemical staining for laminin-γ1 and elastin showed that the BM and elastic fibers of the opposite peritoneum were nearly continuous at 24 hours (Figure 7c). At 4 days, however, the BM of the opposite peritoneum was partly indistinct, and a newly formed BM with a lumen-like structure was observed in the adherent area (Figure 7d). There were more gaps in the peritoneal layer of the elastic fibers at 4 days than at 24 hours, and the lumen-like BM had grown through the gaps in the elastic fibers (Figure 7d). Observation by TEM revealed that blood vessels had grown through the gaps in the elastic fibers of the opposite peritoneum (data not shown).

These observations suggested that the opposite intact tissue participated in tissue repair in the adherent area during fibrous adhesion formation after a cauterized tissue and the opposite intact tissue are approximated.

The outlines of the findings observed in this study are summarized in Table 1.

## Discussion

This is the first study showing that a cauterized tissue causes significant structural changes in the opposite intact peritoneum and is repaired through interaction with the opposite intact tissue, resulting in the formation of a fibrous adhesion. The effects of an injured tissue on an opposing intact peritoneum have not been well studied so far. This is probably because abdominopelvic organs are mobile and it is difficult to locate and observe the opposite peritoneum that was adjacent to the injured tissue in a reproducible manner. Hence, we developed a new model of hepatic adhesions in which two peritoneums always face to each other in the same position. A similar model was developed by Haney and Doty, but the effects of an injured tissue on the opposite intact peritoneum were not investigated in their study^16^. On the other hand, Watters and Buck showed that peeling off MCs without damaging the underlying tissue caused an increase in the number of proliferating MCs on the opposite intact peritoneum at 48 hours^17^. The present study has shown that the deeper injury to the peritoneal surface, which accompanies the necrosis of the underlying tissue, causes a significant change on the opposite intact peritoneum that leads to the formation of fibrous adhesions.

As observed in the present study, the detachment and severe injury of the MCs of the opposite peritoneum until 6 hours leads to loss of the epithelial function of the peritoneum, as indicated by the observation that fibrin attaches to the exposed BM until 24 hours. A negligible increase in the number of inflammatory cells at 1 hour indicates that these cells are not involved in the detachment of MCs at this time. The morphological findings for the opposite peritoneum at 1 hour, such as the denudation in an island-shaped area and the severe injury of MCs at the margin, suggest that the detachment and injury of MCs is caused by a mechanical force, rather than by bioactive substances. MCs have been reported to possess lubricants such as sialomucin or phosphatidylcholine on their apical surface^18^,^19^,^20^,^21^, and the loss of MCs from a cauterized tissue may increase friction between the cauterized tissue and the opposite peritoneum. The accumulation of inflammatory cells associated with the enlargement of the denuded area until 6 hours suggests that inflammatory cells may be involved in further denudation. One possibility is that infiltrated neutrophils injure MCs via oxidative stress^22^,^23^. The exact mechanisms of the denudation of MCs, however, could not be clarified in this study. The early detachment of MCs is in line with observations in a hepatic model of adhesions induced by an intraperitoneal injection of silica and in a pericardial model of adhesions induced by an intrapericardial injection of bacterial toxins, in which intact peritoneums are involved^14^,^24^. The present study shows that an injured tissue causes the detachment of MCs from an opposing intact peritoneum and indicates that the mesothelial detachment is a common step in adhesion formation when intact peritoneums are involved.

It could not be determined whether the morphological changes of MCs into a saccular or a tall shape until 24 hours significantly contributed to the loss of epithelial structure of the opposite peritoneum, given that the denuded area enlarges until 6 hours and appears not to change at 24 hours. The morphological changes are probably distinctive in the opposite peritoneum that comes into contact with the injured tissue because the presence of isolated MCs in a saccular or a tall shape at 24 hours has not been described in other studies on mesothelial regeneration in an injured peritoneum using electron microscopy^25^,^26^,^27^,^28^,^29^. These morphological changes are accompanied by an increase in inflammation, suggesting that inflammatory cytokines or exuded fibrin is involved in the cells' morphological changes. The invariable presence of fibrin in close proximity to the saccular MCs suggests that fibrin is involved in the saccular changes. The integrin isoforms that have been shown to be located at the basal surfaces of MCs might be involved in the interaction between MCs and fibrin^30^,^31^. As to the isolated and tall MCs, the involvement of inflammatory factors is suggested by the reports that the factors change the morphology of cultured MCs into an isolated and cuboidal shape and allow these cells to detach from the underlying matrix^32^,^33^. The isolated and tall MCs are presumed to migrate on fibrin and participate in the epithelialization of the marginal surface of the adherent area at 48 hours. These two types of morphological changes of MCs at 24 hours may enlarge the area of the exposed BM and the fibrin attachment, but the significance of the morphological changes in adhesion formation could not be clarified in this study.

The findings that the denudation of the opposite peritoneum occurs until 24 hours raise a question of how the detached MCs behave afterward. The presence of severely injured MCs entangled in fibrin at 6 hours suggests that some of the detached MCs undergo necrosis. It could not be investigated in the present study whether the detached sheet of MCs until 6 hours is a source of free floating MCs which might serve as a origin of MCs for peritoneal regeneration^34^,^35^. On the other hand, the isolated saccular or tall MCs in the opposite peritoneum at 24 hours might be a possible origin of MCs regenerating the surface of an injured tissue when fibrin is degraded and fibrinous adhesions at 24 hours disappear afterward, that is, the opposite peritoneum might be one of the sources of regenerating MCs for an injured peritoneum^34^,^35^.

In addition to affecting the opposite intact peritoneum, tissue cauterization has been shown to cause tissue repair, to which the opposite intact tissue may contribute. Macrophages play a vital role in tissue repair by releasing growth factors such as TGF-β and VEGF; TGF-β induces the differentiation of myofibroblasts, and VEGF stimulates angiogenesis^36^,^37^,^38^. The significance of macrophages in adhesion formation has been shown by a report that inhibiting the chemotactic aggregation of peritoneal macrophages at the injured site prevents adhesion formation^39^. The accumulation of the cells on the opposite side of the BM/elastic fibers of the opposite peritoneum suggests that the cauterized tissue causes chemotaxis beyond the layer of the BM/elastic fibers. It is not clear what proportion of the accumulated macrophages or myofibroblasts on the cauterized side derives from the opposite side, but the cells may migrate through increasing gaps in the BM/elastic fibers of the opposite peritoneum toward the cauterized side. In contrast, the newly formed blood vessels on the cauterized side obviously derive from the opposite intact tissue. These findings suggest that the opposite intact tissue can provide the adherent and cauterized tissues with the cells that participate in tissue repair and that an intact tissue can cause adhesions when it comes into contact with an injured tissue.

The results of the present study, which found that a cauterized tissue and an opposing intact peritoneal surface together cause fibrous adhesions, are not in line with the findings of another related study. In the study by Haney and Doty, the incidence of adhesions after injury on one uterine horn was compared with the incidence after injury on two uterine horns facing to each other, and the study found that the incidence was notably low after injury on one uterine horn^16^. The apparent difference between the hepatic model and the uterine horn model indicates the need to verify whether adhesions commonly form when an intact peritoneum is involved. Regarding gastrointestinal surgery, the effects of an injured tissue on the opposite intact peritoneum need to be clarified, particularly when injuries are caused at sites that are more relevant to intestinal obstructions, i.e. the intestine, mesentery, and parietal peritoneum^40^,^41^. Moreover, the effects should be investigated when injuries are caused by types of insults other than electrocautery including severe ischemia, which is presumed to be a potent stimulus for adhesion formation^42^,^43^. Any type of insult that frequently causes adhesions between an injured tissue and an intact peritoneum should be minimized in surgical procedures.

The possible role of MCs as a protective epithelium against adhesion formation advocates seeking an effective method of avoiding the detachment and injury of MCs and verifying whether the protection of MCs is associated with adhesion prevention. Prophylactic barrier materials have been developed and shown to be effective in preventing adhesions^6^, yet some experimental studies suggest that commonly available materials are not sufficiently effective^44^,^45^. The results of the present study imply that protecting the MCs of the opposite intact peritoneum leads to the prevention of adhesion formation. However, not much has been reported on the effects of barrier materials on the MCs that lie beneath and in contact with these materials^46^. Barrier materials that maintain the epithelial integrity of MCs in contact and that are not absorbed until the surface of an injured tissue is regenerated by MCs might be the most promising. In addition, as proposed by Haney and Doty, injuries to a normal peritoneum should be minimized wherever the peritoneum may come into contact with the main surgical site where injuries are inevitable^16^. The results of the present study support this view in light of the protective role of MCs. Clarifying what manipulation of a peritoneum causes denudation of the peritoneum and seeking how to prevent the detachment of MCs may contribute to the prevention of adhesion formation.

A limitation of this study is that the methods of investigation are confined to morphological approaches, and an intervention in the model or the quantification of bioactive substances that could affect the function of MCs is needed to further clarify the underlying mechanisms. For example, the involvement of lubricants in the early detachment of MCs may be examined by detecting lubricants on the peritoneal surfaces and verifying whether applying exogenous lubricants, such as sialomucin or phosphatidylcholine, leads to a reduction in peritoneal denudation and the prevention of adhesions^21^,^47^,^48^. The effects of inflammatory cells on the opposite peritoneum at later stages can be investigated by quantifying inflammatory cytokines and verifying whether inhibiting these cytokines leads to the prevention of the morphological changes in MCs. In addition, the exact mechanisms of currently used barrier materials for preventing adhesion formation can be investigated by applying the materials to this hepatic model of adhesions and observing how the materials modulate the effects of an injured tissue on MCs in the opposite intact peritoneum^6^.

In conclusion, the present study shows that an injured tissue affects the opposite intact peritoneum and forms adhesions with the opposite intact tissue. Further studies are needed to clarify the pathophysiology of adhesion formation between an injured tissue and an intact peritoneum. This study supports the significance of mesothelial cells as a protective barrier against adhesion formation and indicates that seeking effective methods of protecting mesothelial cells may contribute to the prevention of adhesion formation and the reduction of adhesion-related morbidity.

## Methods

### Animals

In total, 89 male Sprague-Dawley rats weighing between 300 and 380 g (Charles River Laboratories, Yokohama, Japan) were used. The animals were housed at a constant room temperature with 12-hour light and dark cycles and were provided standard rodent chow and water ad libitum. The research protocol was approved by the institutional animal care and use committee of Asahikawa Medical University. All animal procedures were in accordance with the Guide for the Care and Use of Laboratory Animals (National Research Council of the National Academies).

### Hepatic model of adhesion formation

The medial lobe of the rat liver consists of the right medial lobe (RML) and the left medial lobe (LML)^49^. Adhesions were induced between the RML and the LML, where the two peritoneums are located face to face.

Surgery was performed in an aseptic environment. Rats were anesthetized with diethyl ether. After the skin was shaved and cleansed with 70% ethanol, the abdomen was opened through a 5-cm upper midline incision. The xiphoid process was lifted with a hemostat. The falciform ligament was cut, and two pieces of wet absorbent cotton were inserted beneath the diaphragm to push the liver caudally. The RML was raised with a wet cotton swab, and the exposed medial aspect of the LML was cauterized using bipolar electrocautery forceps (N1-14, Senko Medical Instrument, Tokyo, Japan) by tracing a 1-cm line for 2 seconds (Figures 1a and b). The inner width of the forceps tip was adjusted to 1 mm under a stereomicroscope, and the output of electrocautery (MS-7000E, Senko Medical Instrument) was set at 6 on the dial, 15 watts when measured by an electrosurgery analyzer (RF303, BIO-TEK, Winooski, VT, USA). Physiological saline was dripped onto the cauterized surface to maintain moisture. The medial aspect of the RML, which was opposite to the cauterized aspect of the LML, was grossly observed immediately after cauterization to ensure that no accidental damage on the RML was made by the cautery. The rectus sheath was closed by interrupted sutures, and the skin was closed continuously with 3-0 silk threads.

Relaparotomies were performed at 1, 6, 24, and 48 hours and at 4 and 8 days after cauterization (n = 12 at each time point). As controls, samples were taken from the following groups: normal rats (n = 9); rats immediately after cauterization (n = 4); and rats 24 hours and 4 days after sham operation in which the same procedures were performed except liver cauterization (n = 3 at each time point). The livers were excised and processed for histological examination.

### Preparation for light microscopy

At relaparotomy, the rats were anesthetized with ether and perfused with 20 mL of Ringer's solution containing 10 U/mL heparin, followed by 200 mL of phosphate buffered saline (PBS) containing 4% paraformaldehyde at a flow rate of 10 mL per minute (n = 6 at each time point after cauterization, n = 4 immediately after cauterization, and n = 3 for normal rats and 24 hours and 4 days after sham operation). The medial lobe, containing the RML and LML, was excised en bloc and immersed in the fixative at 4°C overnight.

The RML and LML were cut together in a plain perpendicular to the cauterized line, near the middle of the line (Figures 1b and c). One half of the divided tissue containing the domed portion of the medial lobe was processed for paraffin embedding. The other half of the divided tissue, containing the sharper edges of the RML and LML, was immersed in PBS containing 30% (w/w) sucrose at 4°C overnight and embedded in OCT compound (Sakura Finetek, Tokyo, Japan) using liquid nitrogen.

Next, 5-μm paraffin sections were prepared on a microtome and stained with hematoxylin and eosin (HE). Paraffin sections were used for routine observation and cell counting by immunohistochemical analysis. 8-μm frozen sections were cut on a cryostat and immunostained and are presented in the figures.

### Preparation for transmission electron microscopy (TEM)

Rats were anesthetized with ether and perfused with the Ringer's solution, followed by 200 mL of 0.1 M phosphate buffer (PB) containing 2% glutaraldehyde and 2% paraformaldehyde (n = 3 for normal rats and at each time point after cauterization). The medial lobe was excised en bloc and immersed in the fixative at 4°C overnight.

The RML and LML were cut together in few-millimeter slices perpendicular to the cauterized line, fixed in 0.1 M PB containing 1% osmium tetroxide (Merck) for 2 hours at 4°C, dehydrated in ethanol, substituted with propylene oxide, and embedded in epoxy resin (Epon 812 Resin Embedding Kit, TAAB Laboratories Equipment, Berkshire, UK).

Next, 0.5-μm semithin sections were stained with toluidine blue and examined with an optical microscope. 0.1-μm ultrathin sections were stained with lead citrate and uranyl acetate and examined with a transmission electron microscope (H-7650, HITACHI, Tokyo, Japan).

### Preparation for scanning electron microscopy (SEM)

Rats were anesthetized with ether and perfused with the Ringer's solution, followed by 200 mL of 0.1 M PB containing 2% glutaraldehyde (n = 3 for normal rats and at each time point after cautery). The medial lobe was excised en bloc and immersed in the fixative at 4°C overnight.

When the RML and LML were adherent over a broad area, it was difficult to observe the peritoneums in or adjacent to the adherent area. Therefore, specimens in which the two lobes were separate or loosely attached in a focal area were used for observation. The two lobes were cut apart at the domed portion of the medial lobe. When a loose adhesion was present, an invisible line connecting the two lobes was cut with fine scissors. The tissues were incubated in 0.1 M PB containing 2% tannic acid for 3 hours at room temperature, shaded from the light. After washed, the tissues were incubated in 0.1 M PB containing 1% osmium tetroxide for 2 hours at room temperature, dehydrated in ethanol, substituted with 3-methylbutyl acetate, and dried in a critical point dryer (HCP-2, HITACHI) using liquid carbon dioxide. The tissues were mounted on an aluminum specimen mount (Okenshoji, Tokyo, Japan), coated with platinum-palladium in an Ion Sputter (E-1030, HITACHI), and examined with a scanning electron microscope (S-4100, HITACHI).

### Immunohistochemistry

When antigen retrieval was needed, sections were incubated in pure water containing 0.05% citraconic anhydride (Immunosaver, Nissin EM, Tokyo, Japan) at 95°C for 10 or 45 minutes using a hot water dispenser^50^. The sections were blocked with normal horse serum and incubated with primary antibodies overnight at room temperature. The sections were then incubated with secondary antibodies labeled with Alexa Fluor 488 or 594 (A11029, A11032, A11034, A11037, A11055, and A21207; Life Technologies, Tokyo, Japan) for 1 hour at room temperature. The nuclei were stained with DAPI, and the sections were examined with an optical fluorescence microscope (BX60, Olympus, Tokyo, Japan).

The following primary antibodies were used: anti-keratin (BM555, Acris Antibodies, Herford, Germany), anti-ZO-1 (61-7300, Life Technologies), anti-laminin (L9393, Sigma-Aldrich, Tokyo, Japan), anti-laminin γ1 (sc-59846, Santa Cruz Biotechnology, Santa Cruz, CA, USA), anti-elastin (AB2039, Merck Millipore, Billerica, MA, USA), anti-CD68 (MCA341, AbD Serotec, Bio-Rad Laboratories, Raleigh, NC, USA), anti-α-smooth muscle actin (α-SMA) (M0851, Dako, Tokyo, Japan), and anti-fibrinogen/fibrin (55731, MP Biomedicals, Tokyo, Japan).

### Measurement of the adherent area

The adherent area was measured in paraffin sections stained with HE. The cauterized lobe, i.e. the LML, was positioned downward in the microscopic field, and the opposite lobe, i.e. the RML, was positioned upward, so that the peritoneums were horizontal (Figure 1d). Micrographs were sequentially taken throughout the cauterized area with a ×20 objective lens and were divided by vertical lines into 100-μm-wide sequential segments, which were numbered from left to right in the cauterized area (Figure 1d). The segments in which the two lobes were adherent and the segments in which cauterized hepatocytes were necrotic were counted.

### Counts of MCs and segments in which MCs were absent or discontinuous

MCs were labeled in paraffin sections immunostained for keratin and ZO-1. The two lobes were positioned as described above, and sequential micrographs were taken with a ×40 objective lens, divided into segments, and numbered similarly. The nuclei of the MCs in each lobe or in the adherent area were counted in each segment (Figures 3a and b), and histograms of the counts of nuclei per segment were prepared for all specimens until 24 hours after cauterization or sham operation. In the same sections, the segments in which the MCs of the opposite lobe were absent or discontinuous were counted.

In addition, the nuclei of the isolated MCs in each lobe or in the adherent area were counted in each segment (Figure 3b), and all of the counts of nuclei per segment were added together to calculate the total count of nuclei in the entire section.

### Statistics

Values are expressed as the mean ± s.d. Mann-Whitney's U test was performed by Microsoft Excel (Microsoft Corporation, Redmond, WA, USA).

## Author Contributions

T.K. and T.W. conceived and designed the study. T.S. and H.B. performed the experiments. Y.H. provided technical advice. T.S., H.B., T.K. and T.W. discussed results. T.S. prepared the figures and wrote the manuscript. T.K., T.W. and H.F. reviewed the manuscript.

## Figures and Tables

**Table 1:** Outlines of effects of cauterized tissue on opposite peritoneum (OP)

Time	Mesothelial cells (MCs)	Inflammatory cells and exudate	Cells involved in tissue repair
Im.*	No chages	No changes	No changes
1 h	MCs detach from OP.	No changes	No changes
6 h	MCs detach from OP in a broader area.MCs around denuded area lose microvilli.	Macrophages and neutrophils infiltrate beneath OP.Fibrin attaches to BM of OP.	No changes
24 h	Isolated saccular MCs and tall MCs appear on OP.	Macrophages increase beneath OP.Fibrin attaches to BM of OP in a broader area.	No changes
48 h	Saccular MCs are seen in adherent area.Tall MCs begin to cover marginal surface of adherent area.	Fibrin is seen between two lobes.	Myofibroblasts appear beneath OP.Angiogenesis begins in opposite lobe.
4 d	Tall MCs proceed with regeneration of marginal peritoneum.	Fibrin is replaced by collagen.	Myofibroblasts increase beneath OP.Angiogenesis proceeds through elastic fibers of OP.
8 d	Flat MCs complete regeneration of marginal peritoneum.	No apparent changes	No apparent changes

*immediately after cauterization.
